# Revisiting medial preoptic area plasticity induced in male mice by sexual experience

**DOI:** 10.1038/s41598-017-18248-3

**Published:** 2017-12-19

**Authors:** Arnaud Jean, Pauline Bonnet, Philippe Liere, Sakina Mhaouty-Kodja, Helene Hardin-Pouzet

**Affiliations:** 1 Sorbonne Universités, UPMC Univ Paris 06, INSERM, CNRS, Neuroscience Paris – Seine; Institut de Biologie Paris Seine, 75005 Paris, France; 2 U1195 INSERM and Université Paris Sud and Université Paris-Saclay, 80 rue du Général Leclerc, 94276 Le Kremlin-Bicêtre, France

## Abstract

Sexual experience in male rodents, induced by a first exposure to a receptive female, improves efficiency of following copulations. In mice, the mechanisms supporting this improvement are poorly understood. We characterized molecular modifications of the mouse hypothalamic medial preoptic area (mPOA), the main integrative structure for male sexual behaviour, after a single mating event. This paradigm induced long-lasting behavioural improvements and mPOA morphological changes, evidenced by dendritic spine maturation and an increase in the acetylated and tri-methylated forms of histone H3. Ejaculation affected testosterone, progesterone and corticosterone levels in both naive and experienced mice, but sexual experience did not modify basal plasma or hypothalamic levels of steroids. In contrast to studies carried out in rats, no changes were observed, either in the nitrergic system, or in sex steroid receptor levels. However, levels of glutamate- and calcium-associated proteins, including PSD-95, calbindin and the GluN1 subunit of the NMDA receptor, were increased in sexually experienced male mice. The Iba-1 microglial marker was up-regulated in these animals suggesting multicellular interactions induced within the mPOA by sexual experience. In conclusion, plasticity mechanisms induced by sexual experience differ between rat and mouse, even if in both cases they converge to potentiation of the mPOA network.

## Introduction

Male sexual behaviour is a well-conserved behaviour across several species of rodents. It consists of a motivational phase involving tactile, olfactory and aural interactions with the female, followed by a consummatory phase of bouts of mounting and intromission. This sequence terminates with ejaculation, triggering a refractory period of reduced interest in receptive females^1^. A first exposure to a receptive female leads to a sustainable improvement of this spontaneous behaviour, according to extensive studies in rats. Sexually experienced male rats show an increased sexual motivation characterized by a reduced latency to initiate copulation^2,3^. The improvement of sexual behaviour also concerns the consummatory phase, since sexually experienced rats need less mounts and intromissions, and less time to reach the first ejaculation^4^. Multiple cerebral areas are involved in the control of sexual behavior and mating. They process sensory inputs, regulate rewards and integrate hormonal signals^1^. The medial preoptic area of the hypothalamus (mPOA) is the main integrative site for male sexual behaviour regulation in both rats and mice^5,6^ and its pharmacological disruption leads to the impairment of sexual experience induction in rats^3,7^. Nevertheless, molecular changes underlying improved performance are still poorly understood, particularly in the mouse. In the current study, we thus characterized long-lasting changes in the mPOA induced in the male mouse by a single mating event.

We first focused on the role of sex steroid hormones, since testosterone impregnation is essential for the maintenance of sexual behavior as evidenced by the suppression of copulation by castration and its restoration by hormonal supplementation^1^. Sexual experience is known to increase the baseline^8,9^ or ejaculation-related^10,11^ levels of circulating testosterone in rat. We thus investigated the effects of sexual experience on plasma and hypothalamic post-ejaculation levels of testosterone (T) and its aromatized derivative estradiol (E2) in mice. We also investigated changes in corticosterone (C) and progesterone (P) plasma levels, since their role in male sexual behavior remains unclear^12^. Variations of androgen (AR) and estrogen (ERα) receptors were also studied following sexual experience.

The structural changes induced by sexual experience were investigated by analysing modifications of mPOA dendritic spines. Since learning paradigms are known to induce morphological changes^13^ and increase dendritic spine density in the hippocampus and cortex^14^, the effects of mating and sexual experience were analysed by Golgi-Cox staining and Western blot analysis of associated pre- and post-synaptic markers: synaptotagmin, spinophilin and PSD-95, which have been demonstrated to be involved in the regulation of plasticity processes and dendritic spines stabilization^15–17^.

We also investigated the participation of different neurotransmission systems in the processes underlying sexual learning. We analysed the mPOA nitrergic and glutamatergic systems, since these are known to be impacted by rat sexual status^18,19^. The effect of sexual experience on levels of nNOS and its activity in the mPOA were assessed by Western blot, immunohistochemistry and NADPH-diaphorase staining. Changes in glutamatergic neurotransmission were evaluated by Western blot analysis of proteins, such as pre-synaptic vesicular transporters vGluT1 and vGluT2, NMDA receptor subunits (GluN1, GluN2A and GluN2B), AMPA receptor subunit (GluR2) and calbindin, a dimorphic marker associated with glutamatergic transmission of unclear role in the control of sexual behavior^20^. The effects of sexual experience on glial cells was also investigated since astrocytes and microglia have been implicated in the control of male sexual behaviour^21,22^ and in that of plasticity mechanisms associated with learning^23^. The expression of glial fibrillary acidic protein (GFAP), glutamine synthetase (GS) and glutamate dehydrogenase (GDH) astrocytic markers, and the microglial marker Iba-1 were compared in naive and sexually experienced males by Western blot analysis.

Finally, the level of histone H3 tri-methylated at Lys-27, methylated at Lys-5, and acetylated were also investigated within the mPOA since neuroepigenetic modifications are known to be involved in learning processes^24^ and to play a critical role in male sexual behaviour expression^25,26^.

## Results

### Sexual experience was induced by a single mating

**Figure 1:**
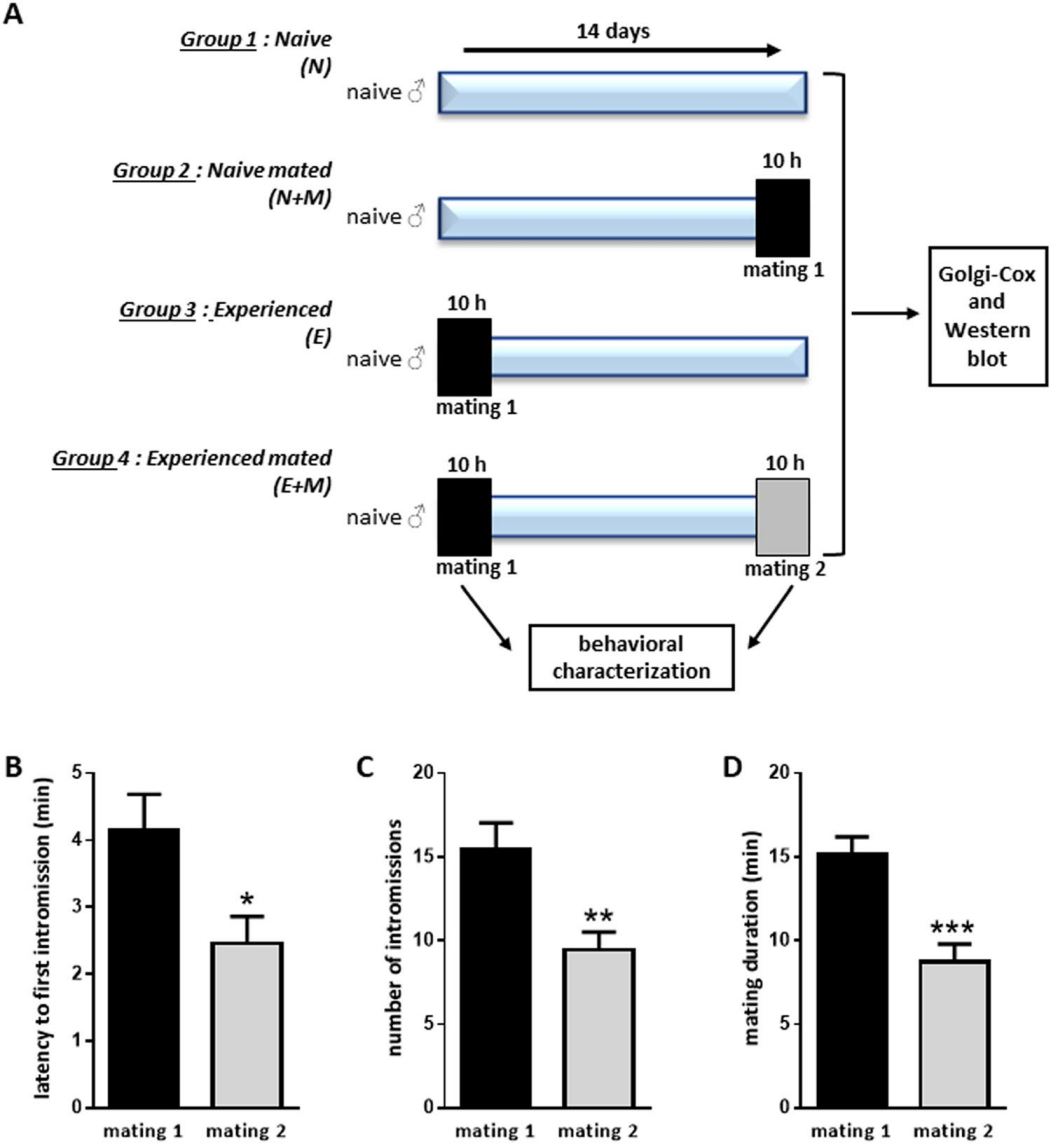
Sexual experience is induced by a single mating. (**A**) Experimental design used to analyse mPOA modifications induced by sexual experience. (N) naive male staying in home cage, (N + M) naive male sacrificed after mating with a receptive female, (E) male mating with a receptive female and sacrificed 14 days later, (E + M) male mated with a receptive female two times, 14 days apart, and sacrificed after the second mating test. Sexual behaviour improvement of (E + M) mice between mating 1 and mating 2 expressed as (**B**) latency to the first intromission, (**C**) number of mounts with intromission, (**D**) mating duration. Results are presented as mean ± SEM (n = 11) and analysed by Student’s test. *p < 0.05, **p < 0.01, ***p < 0.001.

To characterize acquisition of sexual experience, sexual behaviour of experienced animals (E + M) was assessed fourteen days after a single ten-hour exposure to receptive female (Fig. 1A). Analyses of sexual parameters showed a 40% decrease of latency to the first intromission (p < 0.05; Fig. 1B), a 39% decrease of the number of intromissions (p < 0.01; Fig. 1C) and a 42% reduction of the mating duration (p < 0.001; Fig. 1D) indicating the improvement of sexual behaviour 14 days after a unique mating. Moreover, this behavioural improvement was found to persist in a third or fourth mating carried out several weeks later (data not shown). Taken together, these data indicate the relevance of the sexual experience paradigm.

### Ejaculation, but not sexual experience, influenced steroid levels

**Figure 2:**
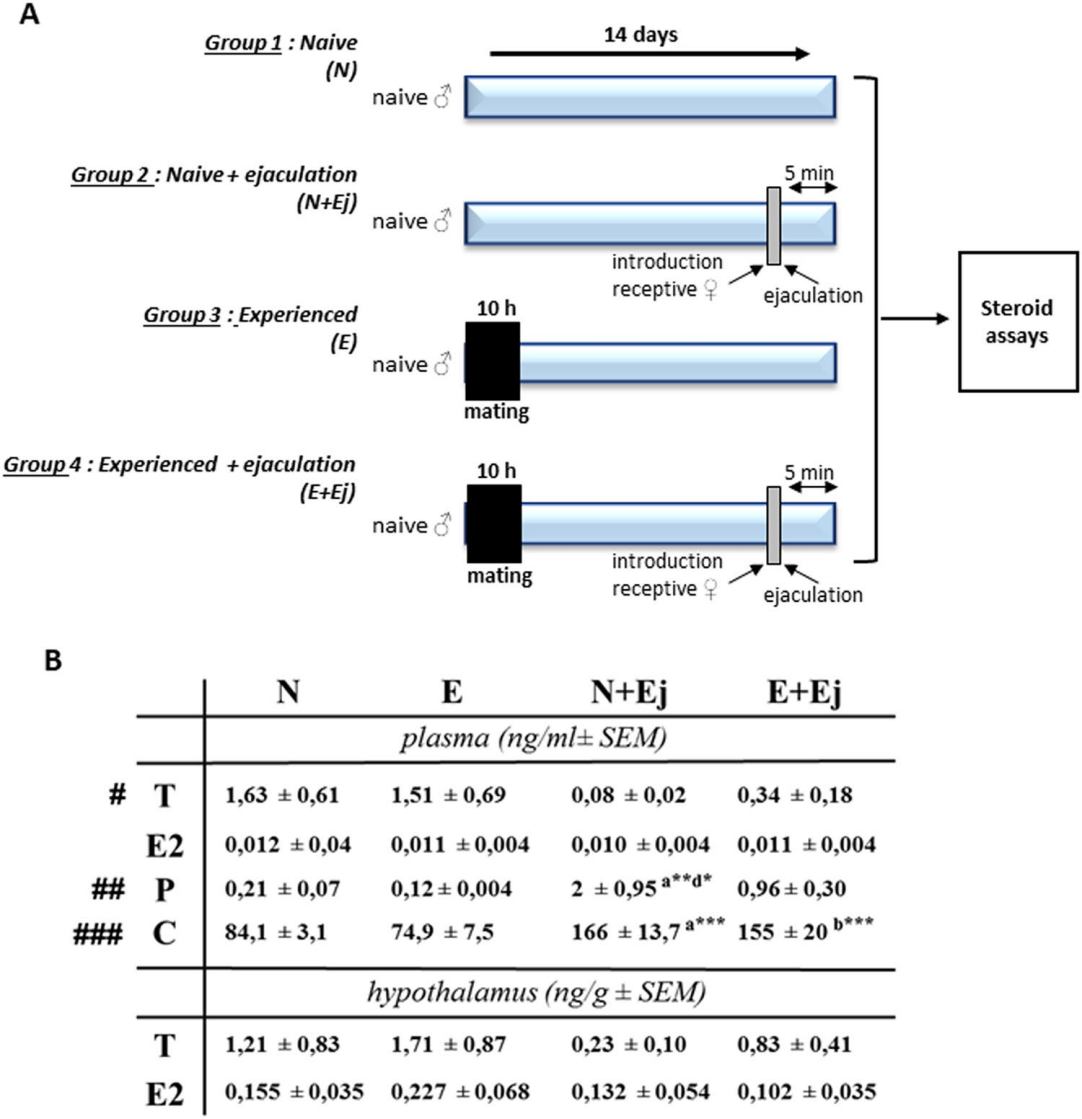
Sexual experience did not change steroid levels within the mPOA. (**A**) Experimental design used to analyse steroid levels in plasma and hypothalamus. N: naive male sacrificed after 14 days in home cage, N + Ej: naive male sacrificed 5 minutes after ejaculating during the first mating, E: male mating 10 h with a receptive female and sacrificed 14 days later, E + Ej: male mating two times 14 days apart during 10 h with a receptive female and sacrificed 5 minutes after the ejaculation of the second mating. (**B**) Plasma and hypothalamic hormonal levels of testosterone (T), 17β-estradiol (E2), progesterone (P) and corticosterone (C). Results are presented as mean ± SEM (n = 4–6 per group) and analysed by two-way ANOVA followed by Bonferroni post-hoc tests, ^#^p < 0.05, ^##^p < 0.01, ^###^p < 0.001 for ejaculation effect, *p < 0.05, **p < 0.01, ***p < 0.001 when compared to N group (a), to E group (b), to N + Ej group (c) or to E + Ej group (d).

Concentrations of hormonal steroids were measured in the plasma and in the hypothalamus of mice from four groups: naive (N), naive just after ejaculation (N + Ej), experienced (E), experienced just after ejaculation (E + Ej) (Fig. 2A).

Two-way ANOVA showed a significant effect of ejaculation on plasma testosterone (decreased, F_(1-20)_ = 5.66, p < 0.05), progesterone (increased, F_(1-20)_ = 8.16, p < 0.01) and corticosterone (increased, F_(1-20)_ = 40.3, p < 0.001) but not on plasma estradiol, hypothalamic testosterone or hypothalamic estradiol. It is interesting to note a tendency to decrease of hypothalamic testosterone after ejaculation, even if this is not significant.

Sexual experience had no effect on plasmat testosterone, estradiol, progesterone, corticosterone, hypothalamic testosterone or hypothalamic estradiol (Fig. 2B).

Post-hoc analysis showed a significant increase in plasma progesterone in the N + Ej group compared to N group (p < 0.01) or E + Ej (p < 0.05). Moreover, plasma corticosterone was increased in the N + Ej group compared to N (p < 0.001) and in the E + Ej group compared to E (p < 0.001). Even not significant, plasma and hypothalamic testosterone concentrations were 4-fold increased in E + Ej group when compared to N + Ej group.

Taken together, these data showed that ejaculation was associated with a reduction of plasma (and hypothalamic) testosterone levels and an increase of both plasma progesterone and corticosterone levels. Sexual experience did not change significantly steroid concentrations.

### Sexual experience did not change steroid receptor levels in the mPOA

**Figure 3:**
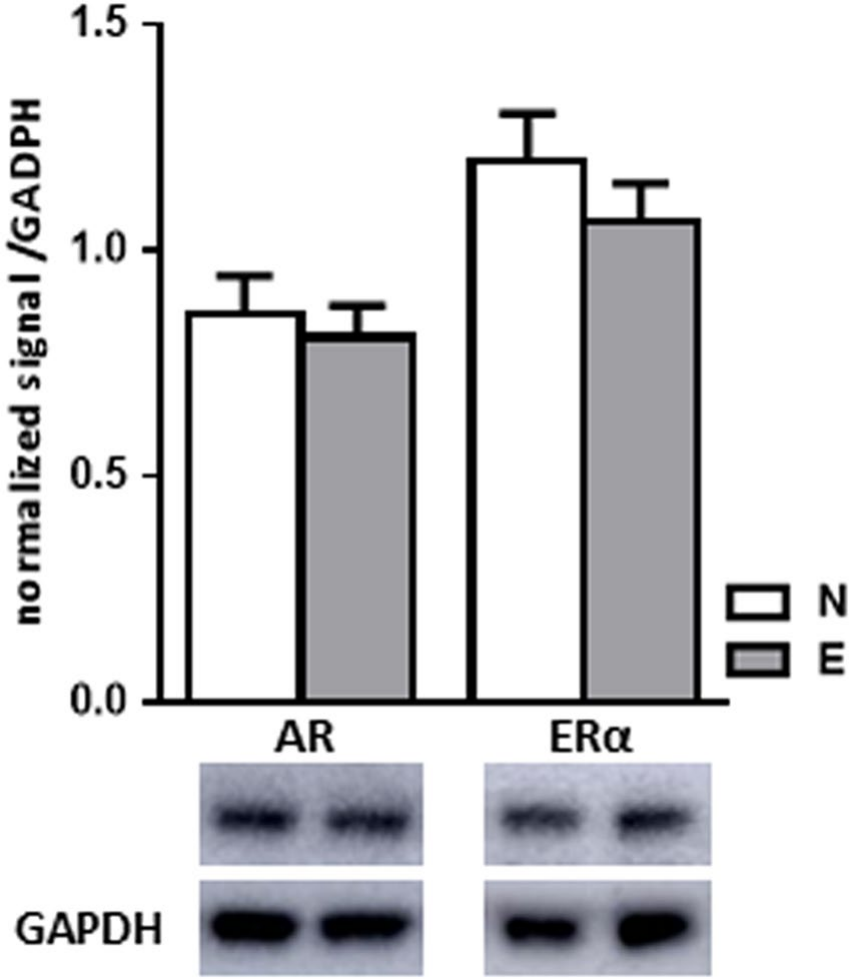
Sexual experience did not change steroid receptor levels within the mPOA. Quantification and representative blot detecting androgen receptor (AR) and oestrogen receptor (ERα) in the mPOA of naive (N) or sexually experienced males (E) (n = 7 mice per group). Results are presented as mean ± SEM and analysed by Student’s test, *p < 0.05 compared to N group. Arrow: mushroom-type spine.

AR and ERα levels were assessed by Western blots in the mPOA of mice from N and E groups (Fig. 3). No significant differences were detected in AR or ERα levels between the two groups. These results indicate that AR and ERα expression in the mPOA are not influenced by sexual experience.

### Sexual experience increased mature dendritic spine density within the mPOA

**Figure 4:**
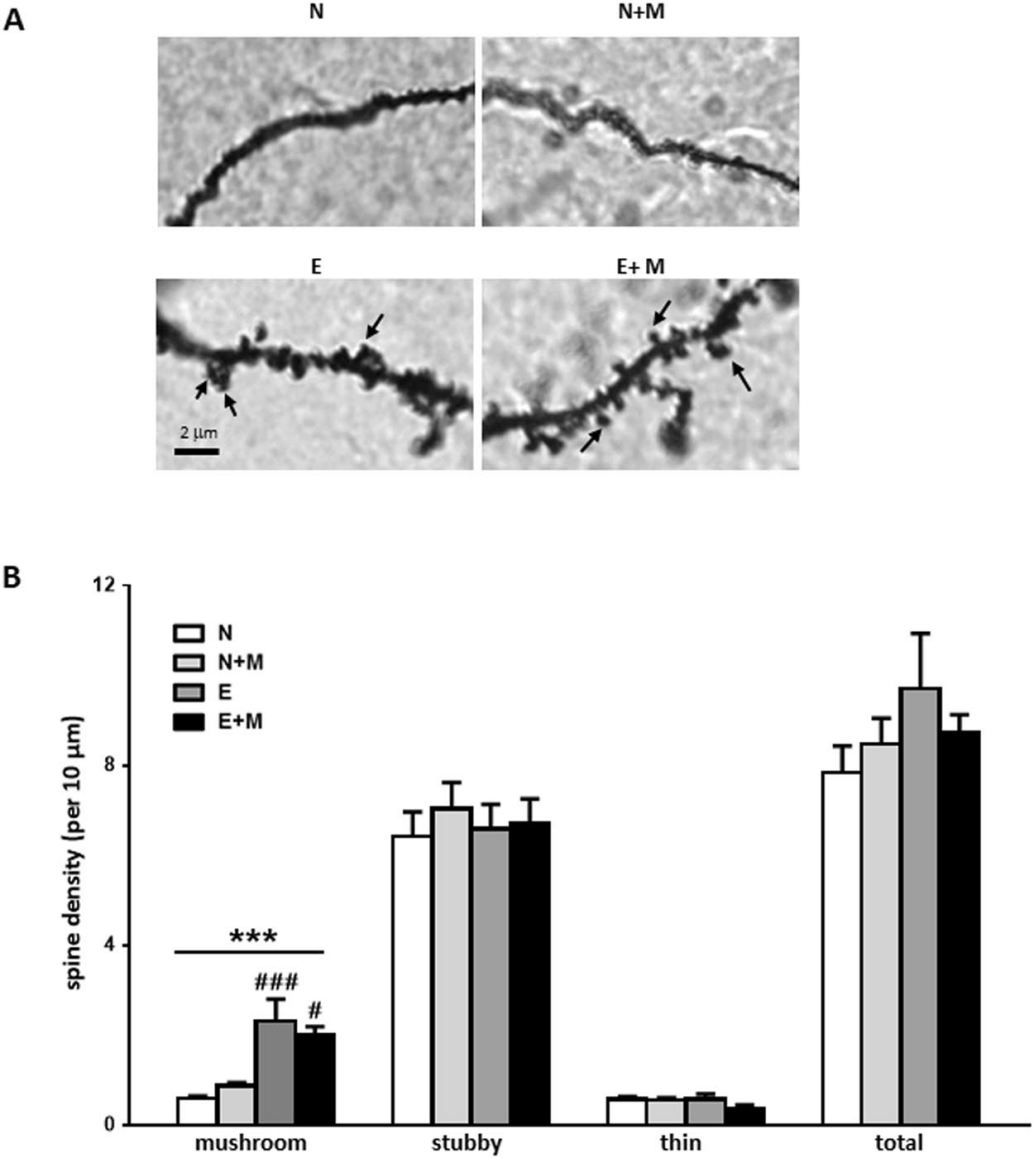
Dendritic spine maturation is induced by sexual experience. (**A**) Representative micrograph of Golgi-Cox impregnated mPOA neurons of naive (N), naive mated (N + M), sexually experienced (E) and sexually experienced mated (E + M) mice. Arrows indicate mushroom dendritic spines. (**B**) Quantification of dendritic spine subtypes (n = 4 mice per group, 10 neurons per mouse). Results are presented as mean ± SEM and analysed by two-way ANOVA followed by Bonferroni post-hoc tests, ***p < 0.001 for sexual experience effect, ^###^p < 0.001 when compared to N group, ^#^p < 0.05 when compared to N + M group.

Golgi-Cox staining was used to characterize dendritic spine density and morphology within the mPOA of naive (N), naive mated (N + M), sexually experimented (E) or sexually experimented mated (E + M) mice (Fig. 4). Dendritic spines were classified in thin, stubby and mushroom according to their maturity level^27^. Morphological observation of the mPOA showed that the number of mushroom spines was increased in experienced groups compared to naive ones. This was confirmed by the quantitative analysis of the density of the different sub-populations in the mPOA. Two-way ANOVA demonstrated a significant effect of sexual experience (F_(1-12)_ = 31.35, p < 0.001) but no effect of mating on mushroom spine density. Post hoc analyses showed an increase in mushroom spine density in the E group (2.32 ± 0.48) compared to N group (0.6 ± 0.05, p < 0.001) and in the E + M (2.04 ± 0.16) group compared to N + M group (0.87 ± 0.07, p < 0.05). In contrast, neither sexual experience nor mating had any effect on stubby, thin and total spine density.

**Figure 5:**
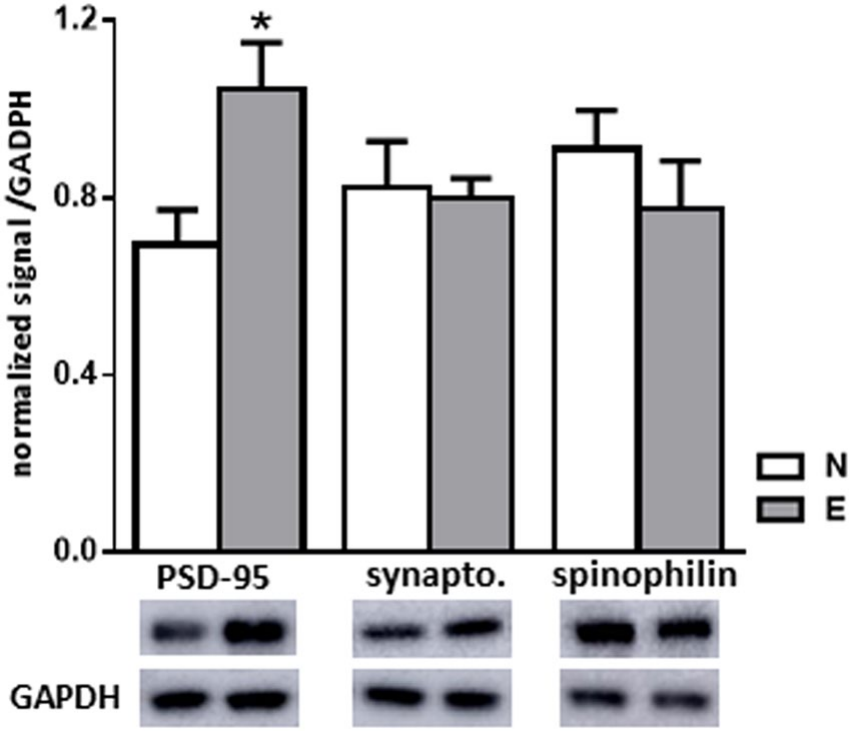
Sexual experience induced increased levels of synaptic markers. Quantification and representative blot detecting synaptic markers in the mPOA of naive (N) or sexually experienced (E) males (n = 7 mice per group). Results are presented as mean ± SEM and analysed by Student’s test, *p < 0.05 compared to N group. synapto = synaptotagmin.

The level of synaptic markers within the mPOA was evaluated for groups N and E (Fig. 5). Western blot analysis showed a 34% increase of the PSD-95 level in group E (p < 0.05) whereas no difference was detected for synaptotagmin or spinophilin.

Taken together, these results showed that sexual experience induced an increase of mature dendritic spine density associated with an increase of the PSD-95 post-synaptic marker within the mPOA.

### Sexual experience did not change nitrergic transmission in the mPOA

**Figure 6:**
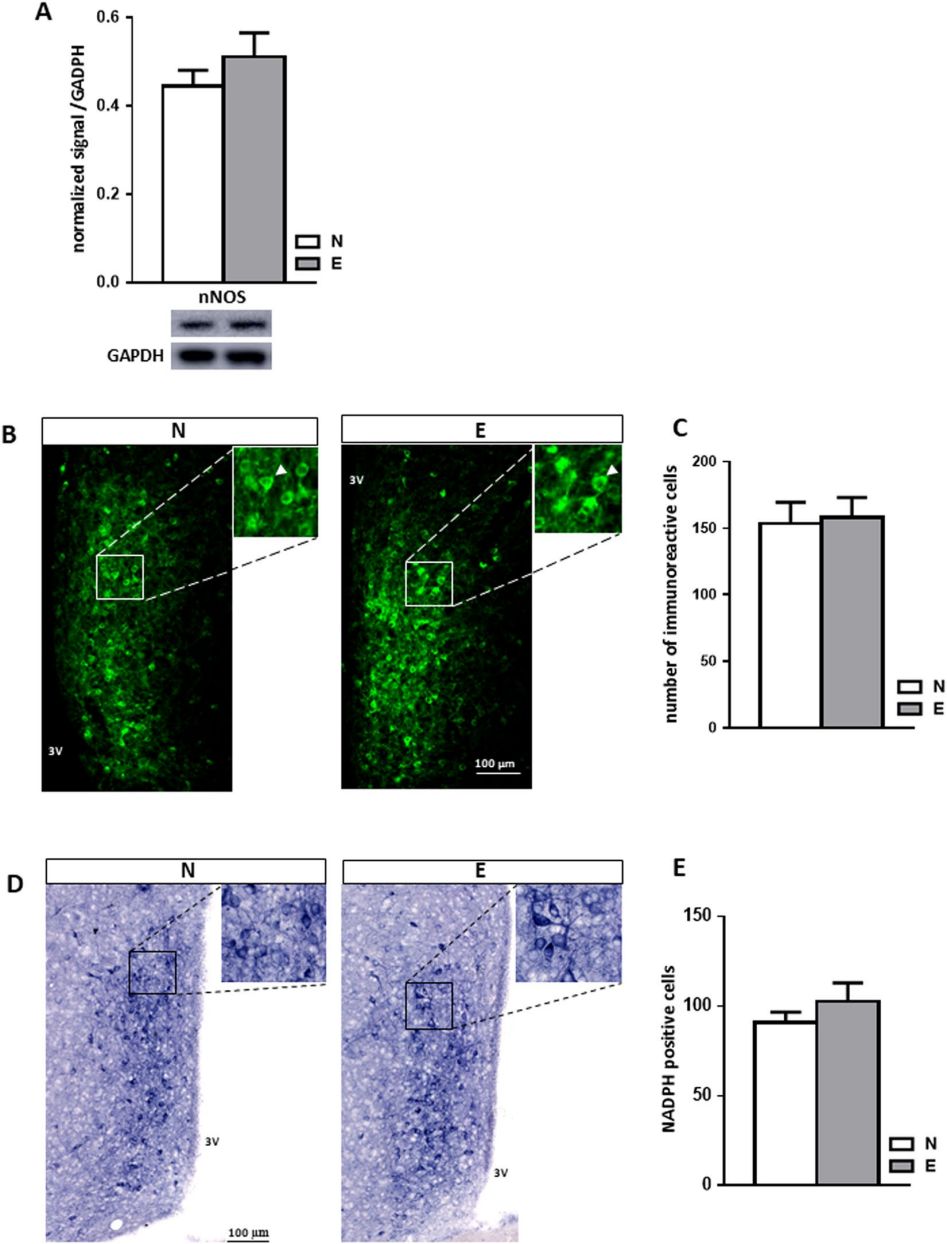
Sexual experience did not change the nitrergic system within the mPOA. (**A**) Quantification and representative blot detecting nNOS in the mPOA of naive (N) or sexually experienced (E) males (n = 7 mice per group). Results are presented as mean ± SEM and analysed by Student’s test. (**B**) Representative IHC detecting nNOS within the mPOA of naive (N) or sexually experienced (E) males (n = 6). (3 V: third ventricule). Arrowhead: positive nNOS cell. (**C**) Quantification of the number of nNOS-expressing cells in the mPOA of naive (N) or sexually experienced (E) males (n = 6 mice per group). Results are presented as mean ± SEM and analysed by Student’s test. (**D**) Representative NADPH-diaphorase staining within the mPOA of naive (N) and sexually experienced (E) males (n = 6). (**E**) Quantification of the number of NADPH-diaphorase positive cells within the mPOA of sexually naive (N) and experienced (E) males (n = 7). Results are presented as mean ± SEM and analysed by Student’s test.

nNOS expression in mice of the N and E groups was evaluated by IHC and Western blot (Fig. 6). The nNOS level assessed by Western blot (Fig. 6A) and the number of cells expressing nNOS (Fig. 6B,C) were both similar between the two groups. NADPH-diaphorase staining was used to evaluate nNOS enzymatic activity. No difference was found between N and E groups concerning the number of cells exhibiting enzymatic activity (Fig. 6D,E). Taken together, these data indicate that sexual experience does not change nitrergic transmission within the mPOA.

### Sexual experience increased glutamate- and microglia-associated protein levels within the mPOA

**Figure 7:**
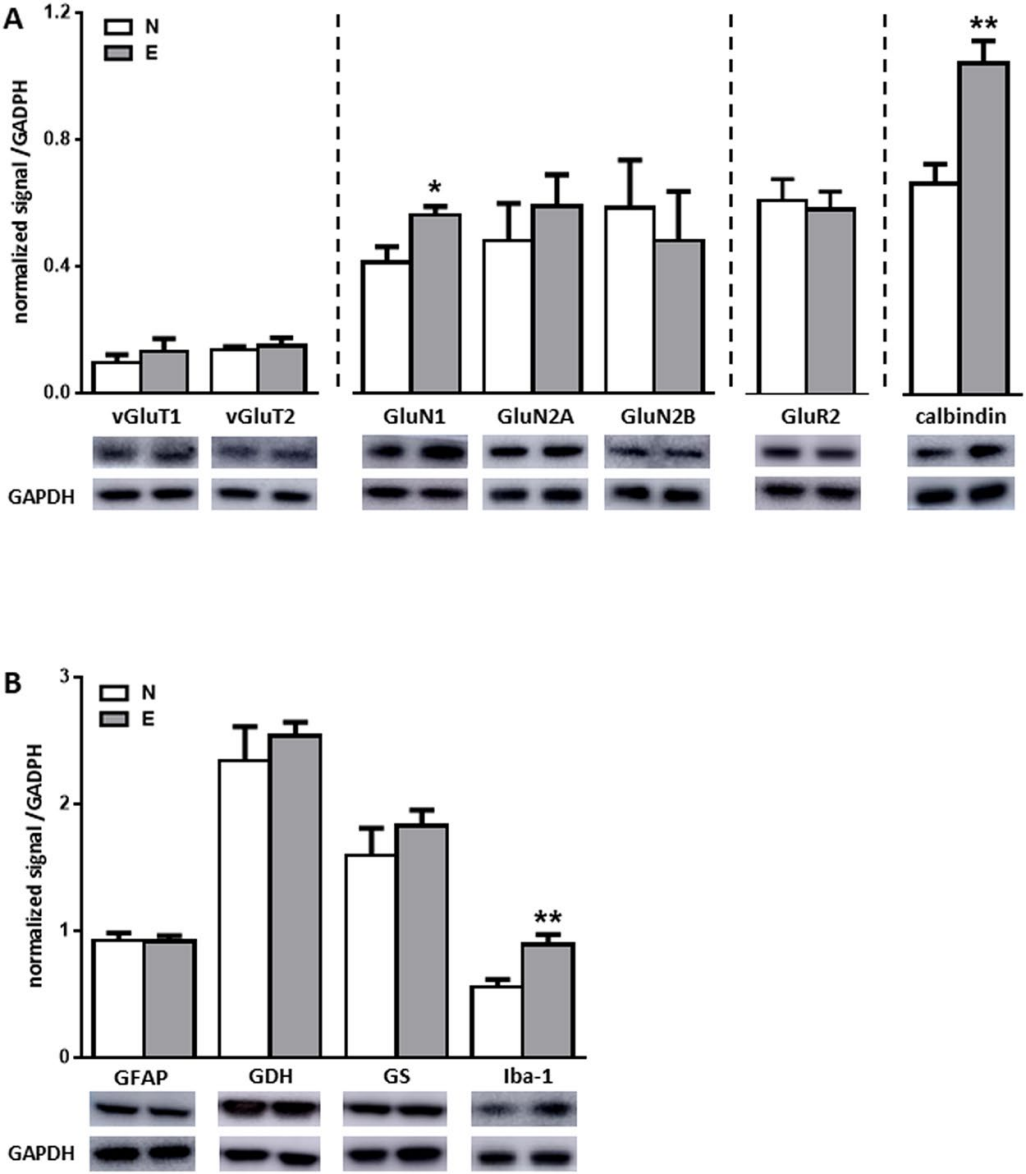
Effect of sexual experience on expression of mPOA glutamatergic-associated proteins and glial proteins. (**A**) Quantification and representative blot detecting glutamate-associated proteins within the mPOA of naive (N) and sexually experienced (E) males. Results are presented as mean ± SEM (n = 7) and analysed by Student’s test. *p < 0.05, **p < 0.01. (**B**) Quantification and representative blot detecting glial markers within the mPOA of naive (N) and sexually experienced (E) males. Results are presented as mean ± SEM (n = 7) and analysed by Student’s test, **p < 0.01.

The level of glutamatergic-associated markers in N and E group mice was evaluated by Western blot (Fig. 7A). Pre-synaptic vGluT1 and vGluT2 were similar for mice of both groups. The GluN1 level was significantly increased in E group mice compared to those of N group (0.41 ± 0.05 *vs*. 0.56 ± 0.03; p < 0.05) and no difference was seen for other NMDA subunits such as GluN2A, GluN2B or the obligatory AMPA subunit GluR2. Sexually experienced mice also exhibited higher levels of calbindin, a protein involved in plasticity and calcium buffering (0.66 ± 0.06 *vs*. 1.0 ± 0.07; p < 0.01). No change in the number of cells expressing calbindin was detected (data not shown), suggesting an increase in the intracellular level of this protein.

The levels of the astrocytic markers GFAP, GDH and GS were unchanged in contrast to the microglial marker Iba-1, which was significantly increased after sexual experience (0.55 ± 0.06 *vs*. 0.89 ± 0.08; p < 0.01) (Fig. 7B), even if the number of Iba-1 positive cells was unchanged (data not shown).

Taken together, these results indicate that sexual experience was associated with increased levels of the NMDA subunit GluN1 and calbindin in the mPOA. It also raised the Iba-1 levels, suggesting an involvement of microglia in the processes underlying sexual experience.

### Sexual experience induced epigenetic modifications within the mPOA

**Figure 8:**
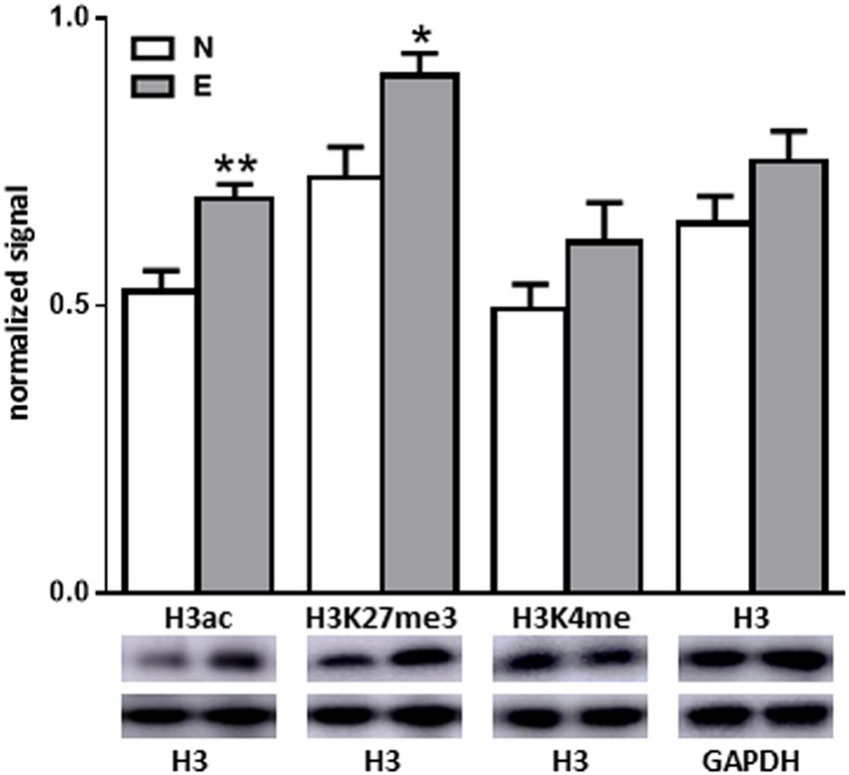
Sexual experience induced epigenetic modifications. Quantification and representative blot detecting acetylated H3 (H3ac), H3 trimethylated at Lys-27 (H3K27me3), H3 methylated at Lys-4 (H3K4me) and total H3 levels. H3K27me3 and H3K4me signals were normalized to H3 whereas H3 levels were normalized to GAPDH. Results are presented as mean ± SEM and analysed by Student’s test, *p < 0.05 compared to N group.

Long-lasting epigenetic changes induced by sexual experience were assessed by comparing the levels of acetylated H3 (H3ac), H3 trimethylated at Lys-27 (H3K27me3) and H3 methylated at Lys-5 (H3K4me) between N and E groups (Fig. 8). Western blot analysis indicated a significant 30% increase of H3ac (p < 0.01) and a significant 25% increase of H3K27me3 levels (p < 0.05) in E groups, whereas no difference was detected for H3K4me or H3 levels. These results established that sexual experience induced epigenetic changes detectable 14 days after the first mating.

## Discussion

The sexual behavioural template is organized successively in motivational and copulatory phases and is subjected to learning processes. Sexual performances of a previously mated male rodent are increased compared to a naive one. Most studies have induced sexual experience by long exposure to receptive female^28,29^. In this article, we assess the induction of sexual experience by a single mating event, by comparing the sexual parameters of naive males allowed to copulate twice, separated by fourteen days. A single 10 h exposure to a receptive female was sufficient to induce an amelioration of male mice sexual behavior. Indeed, males then showed a decreased latency to initiate copulation and a reduced mating duration in their second mating.

The medial preoptic area of the hypothalamus (mPOA) is considered as the main integrative site for male sexual behaviour regulation^30^. Little is known about mPOA molecular changes during the acquisition of sexual experience, especially in the mouse. In this work, we investigated the modifications of the mouse mPOA triggered by this single mating event.

In the rat, the nitrergic system is known to be massively involved in the central control of sexual behaviour and in the processes underlying induction of sexual experience^7,18^. Our results demonstrated that neither the number of nNOS-positive cells, nor the level of nNOS protein were modified by sexual experience. Furthermore, the enzymatic activity of nNOS, estimated *via* NADPH-diaphorase staining, was similar in naive and sexually experienced male mice. All these results strongly contrast with those described in rats^18,31^. They suggest that the processes leading to sexual experience are different between the two species, and involve distinct neurochemical pathways in the mouse.

The hormonal status of naive against sexually experienced mice was characterized in basal conditions or immediately after ejaculation. Levels of testosterone decreased just after ejaculation in both groups of mice in plasma and in a less extent in the hypothalamus. This contrasts with results obtained in rats by Kamel *et al*.^11^ and Edinger and Frye (2007)^8^ and could be related to behavioural differences between species: rats are able to ejaculate multiply whereas mice have a single ejaculation^1^. Estradiol levels were unaffected by ejaculation, but we showed here for the first time that both progesterone and corticosterone were increased just after ejaculation. The elevated levels of corticosterone remained within a physiological range^10,32^, indicating the absence of stress related to the experimental paradigm: this corticosterone elevation would rather be connected to the increased activity of the male during encounter with a female. We measured the concentration of testosterone and estradiol in the hypothalamus for the first time in mouse. Our results don’t support an increase of the aromatase activity in the hypothalamus in response to sexual activity. Hypothalamic testosterone levels are in equilibrium with plasma levels with lower values after ejaculation and estradiol levels were unchanged by sexual experience and ejaculation. This is in contrast to changes in aromatase enzymatic activity shown in the quail mPOA^33^. Moreover, the comparison between naive (N) and males sacrificed 14 days after the first mating (E) indicated similar levels of androgen (AR) and estrogen (ERα) receptors within the mPOA, confirming data of Wu and Gore (2009)^9^. These results are consistent with the similar testosterone concentration we found in both naive et sexually experienced mice, since AR expression is known to be regulated by testosterone levels^34^. In summary, our results indicate that in mice, testosterone impregnation is necessary for sexual experience induction, rather than the molecular support triggering the behavioural improvement. This is sustained by castration-supplementation experiments, which ensure a stable testosterone impregnation, while still allowing sexual experience acquisition^35^.

Behavioural improvement induced by sexual experience was accompanied by structural and molecular modifications of the mPOA. The dendritic spine micro-architecture visualized by Golgi-Cox staining revealed a specific rise in the number of mature mushroom spines in sexually experienced mice. Such an increased spine density was associated with male sexual behaviour retention following castration in mice^36^, and has already been demonstrated to be induced by sexual experience in the hippocampus and prefrontal cortex of male rats^37^. This maturation process of dendritic spines towards a mushroom phenotype, is characteristic of learning processes^14^ and is associated with modifications of synaptic strength^13^. Functional consequences of the dendritic spine modifications in the mPOA were assessed by a panel of synaptic markers: synaptotagmin, a pre-synaptic Ca^2+^ sensor involved in exocytosis^38^, spinophilin, a post-synaptic protein known to be dimorphically expressed in rat^39^ and known to positively correlate with male sexual behaviour^40^, and PSD-95, a post-synaptic protein found in excitatory synapses whose level correlated with an increased size of dendritic spines^41^. Levels of PSD-95 were increased by sexual experience. Since PSD-95 is involved in the synaptic membrane localization of NMDA and AMPA glutamate receptors, our data are in favour of an enhancement in the sensitivity of the excitatory network in sexually experienced mice^42^.

The absence of dendritic modification the day after the first mating suggests the existence of a maturation period. This hypothesis is supported by the higher levels of acetylated and trimethylated H3 found in the mPOA of sexually experienced males that demonstrate epigenetic regulations triggered by sexual experience. Trimethylation at Lys-27 of histone H3 (H3K27me3) is known to induce chromatin compaction and inhibition of gene transcription^43^, whereas H3 methylation at Lys-5 (H3K4me) enhance gene transcription^44^. Several gene directly involved in dendritric spine maturation, such as PP1 and calcineurin, have been evidenced to be targeted by epigenetic processes^24^. Other pathways could also be affected, particularly those involved in the control of sexual behaviour, as it has been demonstrated during the establishment of sex differences in the brain^26^. Finally, the kinetic of the long-term stable epigenetic molecular changes may explain why performance improvement required a 14 days interval to become established and to remain definitive, regardless of the number of male mouse copulations.

As suggested by the increase in PSD-95 and GluN1 levels we found in sexually experienced males, the mPOA glutamatergic network could be one of the cerebral systems involved in the behavioural improvement induced by sexual experience in mice^19^. The release of glutamate by afferent terminals in the mPOA seemed to be unaffected by sexual experience, since no modifications in the levels of vGluT1 or vGluT2 were detected. In contrast, sexually experienced mice exhibited higher levels of GluN1. This is consistent with the increase of the phosphorylated form of GluN1 detected after mating within the rat mPOA^45^. Despite the absence of detectable changes in the level of other glutamate receptor subunits, these may be involved in plasticity mechanisms associated with sexual experience *via* the regulation of their subcellular localization as it has already been shown in the rat mesolimbic area^46^. Our results suggest that the processes of sexual learning involve NMDA sensitivity of the mPOA glutamatergic network. This conclusion is consistent with several observations: NMDA receptor upregulation is known to be stimulated by an increase in glutamate release^47^, and nearly 100% of cells expressing mating-induced c-Fos also express the GluN1 subunit within the mPOA^45^. Furthermore, increased NMDA current triggered by sexual experience, as shown in the rat mesolimbic area^48^, was associated with increased calcium intracellular levels. This is in agreement with the increased levels of calbindin that we found in sexually experienced mice, since this protein is known to act as an intracellular calcium buffer and to be involved in synaptic plasticity mechanisms^49^.

In the last part of our work, we wanted to examine the involvement of non-neuronal cells in the mechanisms underlying sexual experience, since astrocytes and microglia are known to be involved in regulation of glutamatergic neurotransmission and spinogenesis^50^. GS, GDH and GFAP expression were not modified by sexual experience, confirming data obtained by Will *et al*.^51^ showing that the number of astrocytes did not differ within the mPOA between naive and sexually experienced rats. By contrast, we observed an increased level of Iba-1, the ionized calcium binding adaptor molecule 1, in sexually experienced mice. An increasing number of studies characterized the involvement of microglia in neurotransmission regulation and its role in non-pathological brain plasticity mechanisms^49,52^. Microglial cells are capable of detecting neuronal activity via the expression of a large variety of neurotransmitter receptors^53^ and to directly interact with synapses^54^. Microglia also releases substances such as BDNF known to modulate synaptic plasticity and NMDA receptor properties^55^. Moreover, in the mPOA, the density and branching of microglia, which are higher in the male than the female, regulate the microarchitecture and dendritic spine density in the developing brain^56^. It has also been shown that adult brain depletion of microglia disrupted learning processes and dendritic spine formation, and modified the glutamatergic synapse composition in glutamate receptor subunits^23^. These data associated with our results suggest that microglia could actively participate in plasticity mechanisms within the adult mPOA triggered by sexual experience.

In conclusion, our data showed for the first time that one mating was sufficient to induce behavioural improvement and long-lasting morphological, molecular and epigenetic changes in the mPOA. Contrary to data obtained in rat, steroid concentrations were not affected by sexual experience, confirming that sex hormones, including testosterone, play instead a permissive role in establishing mPOA changes related to sexual experience in mouse. The first female encounter by a naive male mouse led to an increase in the sensitivity of the glutamatergic network of the mPOA, assessed by the increased levels of GluN1 and calbindin. Contrary to data obtained in rat, these changes did not involve an increase of nNOS expression in mouse but probably involved the participation of microglia, as shown by the increased levels of Iba-1. Although these results are in opposition to several findings described in rats, sexual experience resulted in a similar consequence: increased sensitivity of the mPOA in sexually experienced males that would accelerate the progression of the successive phases of sexual behaviour, leading to a reduction of the mating duration.

More generally and from a developmental point of view, the plasticity mechanisms highlighted in this study allow the acquisition of sexual experience to be considered as an ultimate phase of maturation of the cerebral structures controlling sexual behavior. This third phase, after the perinatal and pubertal phases, permits the expression of a fully mature sexual behaviour with structural and molecular changes involving some of the same actors, such as dendritic spine, glutamate and microglia that are already involved in perinatal dimorphism and sexual differentiation^56^.

## Methods

### Animals

All experiments were conducted according to French and European laws (Decrees 2013-118, L214 and R214-87/130, 2013/63/ECC) and approved by the “Charles Darwin” ethical committee (project #01490-01).

C57Bl/6 J mice (Janvier Breeding Centre, Le Genest, France) were bred in our animal facility and housed under a controlled photoperiod (12 h light and 12 h darkness cycle - lights on at 2 a.m.) at 20 ± 2  °C with food and water *ad libitum*. Ten weeks old adult males were isolated for two weeks before experiments.

### Preparation of receptive females

C57BL/6 J females were ovariectomized under general anaesthetic (xylazine 10 mg/kg - ketamine 100 mg/kg, i.p.) and implanted with Silastic (Dow Corning) implants filled with 50 μg of estradiol-benzoate (Sigma-Aldrich, Saint-Louis, USA) in 30 μl of sesame oil. Four hours before tests, they were subcutaneously treated with 1 mg of progesterone (Sigma-Aldrich, Saint-Louis, USA) in 100 μl of sesame oil^35^. Female receptivity was verified before the beginning of experiments with a sexually experienced male.

### Male sexual behaviour

Males were assigned to one of four different groups (Fig. 1A): naive male staying in home cage (Naive: N), naive male allowed to mate once for 10 h with a receptive female (Naive mated: N + M), naive male mated once with a receptive female for 10 h and then kept in home cage for 14 days (sexually experienced: E) and naive male mated once with a receptive female for 10 h, then kept in home cage for 14 days and allowed to mate a second time for 10 h (sexually experienced mated: E + M).

Tests were carried out under red-light illumination 2 h after lights were turned off and videotaped for further analyses. After 4 h of habituation, each male was tested in its home cage for 10 h after the introduction of a receptive female. Male sexual behaviour was analysed by scoring the latency to the first intromission, the number of mounts with intromissions and the mating duration defined as the time from the first intromission to ejaculation.

Males of each group were randomly assigned to Golgi-Cox staining (4 per group). N and E males were used for Western blot analysis (7 per group), IHC experiment (6 per group) or NADPH-diaphorase staining (7 per group).

### Golgi-Cox staining

Male mice were decapitated, and their brains rapidly removed and fixed for 10 days in Golgi-Cox solution (NovaUltra Golgi-Cox Stain kit, Interchim, Montluçon, France). Coronal slices were cut with a vibratome (Leica Microsystems VTC1000S, Wetzlar, Germany). Using the anterior commissure as a landmark, 30 µm-thick hypothalamic sections including the mPOA were immersed in post-impregnation solution for 10 minutes, washed in PBS and mounted with Mowiol. For each animal, 10 neurons homogeneously distributed throughout the mPOA were analysed. The number of dendritic spines were normalized to 10 µm segments. Subtypes of dendritic spines were determined by applying Harris criteria^57^.

### Immunohistochemistry

Animals were deeply anesthetized with sodium pentobarbital (150 mg/kg, i.p.) and perfused with 4% paraformaldehyde (PFA) in 0.1 M phosphate buffer (PB). After 4 h post-fixation, 30 μm coronal sections were cut as mentioned above and incubated for 2 h in PBS containing 1% bovine serum albumin (BSA; Sigma-Aldrich, Saint-Louis, USA) and 0.2% Triton X-100 and then incubated for 24 h at 4 °C with primary antibodies against nNOS (1:1000, produced in goat, Abcam, Cambridge, UK). Slices were then incubated for 2 h with Alexa Fluor 647-conjugated donkey anti-goat secondary antibody (1:1000; Thermo Fisher Scientific, Waltham, USA). They were then mounted with Mowiol. The number of nNOS immunoreactive cells were counted bilaterally on two areas of 0.8 mm^2^ located on the same section within the mPOA. Anatomically matched sections were identified using the Mouse Brain Atlas of Paxinos, plate 30^58^.

### NADPH-diaphorase staining

Animals were perfused as outlined above and 30 µm-thick frozen floating sections (Leica CM 3050 S, Wetzlar, Germany) were obtained. NADPH-diaphorase enzymatic activity was measured using the reduction of 0.2 mM nitroblue tetrazolium (NBT, Sigma-Aldrich, Saint-Louis, USA) with 1.2 mM β-NADPH (tetrazolium salt, Sigma-Aldrich, Saint-Louis, USA) in Tris buffer pH 8, 0.2% Triton X-100 at 37 °C for 2 h. Sections were mounted with Permount (Thermo Fisher Scientific, Waltham, USA). The number of NADPH-diaphorase positive cells were counted bilaterally on two areas of 0.8 mm^2^ located on the same section, as described above.

### Western blot analysis

The brains of decapitated male mice were sliced with a Vibroslice (World Precision Instrument, Sarasota, USA) in cold 0.1 M PB. One 400 µm-thick section including the mPOA was punched out. Samples were homogenized in 50 mM Tris (pH 7.2), 150 mM NaCl, 0.1% SDS, 0.5% sodium deoxycholate, 10 mM EDTA, 10 mM EGTA, 1% Triton-X100 and 1% protease Inhibitors (Roche, Meylan, France) before being processed for Western blot analysis as described^59^. Blots were incubated overnight with primary antibodies (Table 1). To determine the level of protein expressed, the protein signal was normalized to the GAPDH or H3 signal.

**Table 1:** Primary antibodies used for Western blot analysis.

Protein	dilution	species	manufacturer
Ac H3	1:1000	rabbit	Millipore, Guyancourt, France
AR	1:300	rabbit	Santa Cruz Biotechnology, Dallas, USA
Calbindin	1:2000	mouse	Sigma-Aldrich, Saint-Louis, USA
ERα	1:300	rabbit	Santa Cruz Biotechnology, Dallas, USA
GAPDH	1:20 000	mouse	Santa Cruz Biotechnology, Dallas, USA
GDH	1:1000	rabbit	Interchim, Clichy, France
GFAP	1:2000	mouse	Sigma-Aldrich, Saint-Louis, USA
GluN1	1:500	mouse	Millipore, Guyancourt, France
GluN2A	1:500	rabbit	Millipore, Guyancourt, France
GluN2B	1:300	mouse	Abcam, Cambridge, UK
GluR2	1:750	mouse	Millipore, Guyancourt, France
GS	1:2000	mouse	BD Transduction Laboratories, Franklin Lakes, USA
H3	1:1000	rabbit	Abcam, Cambridge, UK
H3K27me3	1:1000	mouse	Diagenode, Liege, Belgium
H3K4me	1:1000	rabbit	Diagenode, Liege, Belgium
Iba-1	1:750	rabbit	Wako Chemicals, Richmond, USA
nNOS	1:500	mouse	Sigma-Aldrich, Saint-Louis, USA
PSD-95	1:500	mouse	Santa Cruz Biotechnology, Dallas, USA
spinophilin	1:1000	rabbit	Abcam, Cambridge, UK
synaptotagmin	1:1000	goat	Santa Cruz Biotechnology, Dallas, USA
vGluT1	1:4000	rabbit	Abcam, Cambridge, UK
vGluT2	1:3000	rabbit	Abcam, Cambridge, UK

### Steroid assay

Mice were assigned to one of four groups (6 males per group) (Fig. 2A): naive males staying in home cage (Naive: N), naive males allowed to mate once and sacrificed 5 minutes after ejaculation (Naive + ejaculation: N + Ej), naive male mating for the first time for 10 h and then kept in home cage for 14 days (Experienced: E) and naive males mating for the first time for 10 h, kept in home cage for 14 days, allowed to mate a second time and sacrificed just after ejaculation (Experienced + ejaculation: E + Ej). Males were decapitated, the plasma was collected and the hypothalamus was dissected out and frozen at −80 °C. Steroids were extracted from the plasma (200 µl) and hypothalamus (5–10 mg) using methanol. Samples were purified and fractionated by solid-phase extraction as previously described^60^. The unconjugated steroid-containing fraction was filtered, purified and fractionated by HPLC. Two fractions were collected: testosterone, estradiol and progesterone were eluted at 13–25 minutes and were derivatized with 25 µl heptafluorobutyric anhydride (HFBA) and 25 µl anhydrous acetone (1 h, room temperature). Corticosterone was eluted at 25–33 minutes and derivatized (1 h, 80 °C) with 25 µl HFBA and 25 µl anhydrous acetonitrile^61^. The fractions were dried for GC-MS/MS analysis using an AI 1310 autosampler, a Trace 1310 gas chromatograph (GC), and a TSQ 8000 tandem mass spectrometer (MS/MS) (Thermo Fisher Scientific San Jose, CA) using Argon as the collision gas. The identification of steroids was supported by their retention time and dependence of two or three transitions. Quantification was performed according to the major ion produced, using a previously established calibration curve^62^.

### Statistical analysis

Statistical analysis was performed using Graphpad Prism software (GraphPad Software, La Jolla, California, USA). Data were expressed as mean ± SEM. Sexual improvement was analysed with paired Student’s test. Two-way ANOVA followed by Bonferroni tests were used to analyse the effects of mating and sexual experience on dendritic spine morphology and sex steroid changes. Other data were analysed with Student’s test. *p* values of less than 0.05 were considered significant.

## Electronic supplementary material

Supplementary information
