# Supplementary material for: Revisiting medial preoptic area plasticity induced in male mice by sexual experience
Source: Sci Rep. 2017 Dec 19;7:17846. doi: 10.1038/s41598-017-18248-3 (PMC5736590; doi:10.1038/s41598-017-18248-3)
Supplement: Supplementary file 1 — Supplementary information [file 41598_2017_18248_MOESM1_ESM.pdf]

Supplementary information for the manuscript SREP-17-41974A :

Revisiting medial preoptic area plasticity induced in male mice by sexual experience

Arnaud JEAN, Pauline BONNET, Philippe LIERE, Sakina MHAOUTY-KODJA and Hélène HARDIN-POUZET*

AR (0.8 sec exposition)

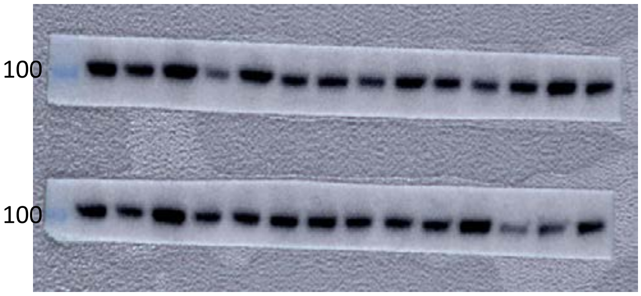

GAPDH (0.1 sec exposition)

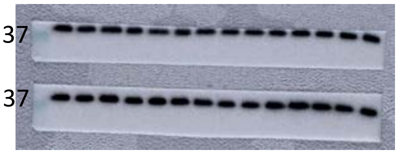

Sample order :
top : PM-N * E * N * E * N * E * N E
bottom : PM-N * E * N * E * N * E * * *

ERα (3 sec exposition)

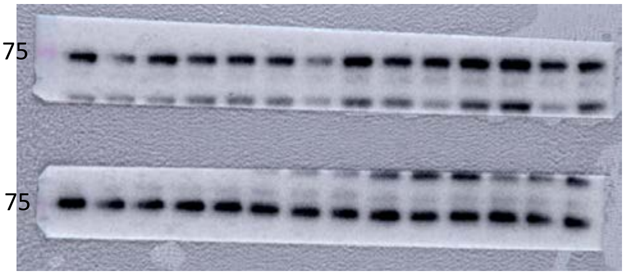

GADPH (1 sec exposition)

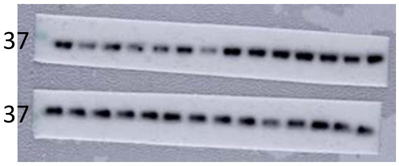

Sample order :
top : PM - N * E * N * E * N * E * N E
bottom : PM - N * E * N * E * N * E ***

PSD95 (4 sec exposition)

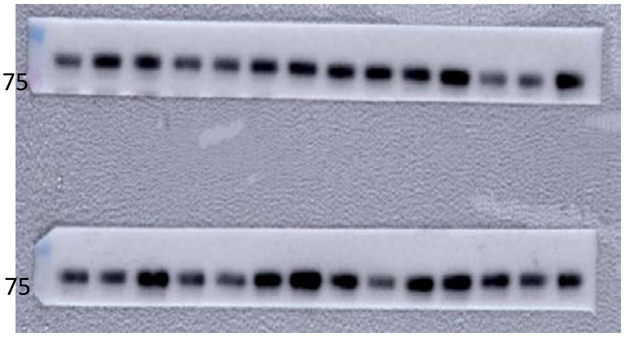

GAPDH (0.5 sec exposition)

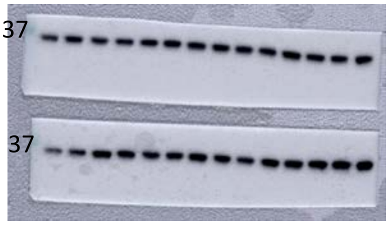

Sample order :
top : PM - N * E * N * E * N * E * N E
bottom : PM - N * E * N * E * N * E ***

Synaptotagmin (2 sec exposition)

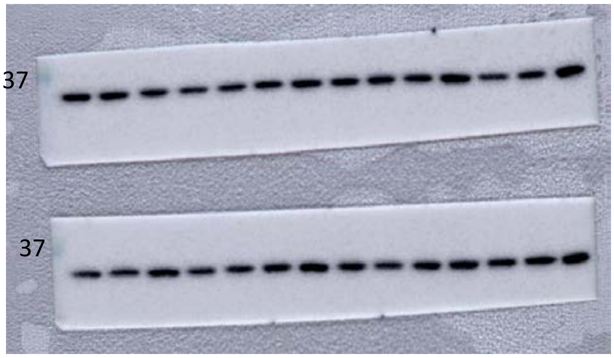

GAPDH (0.5 sec exposition)

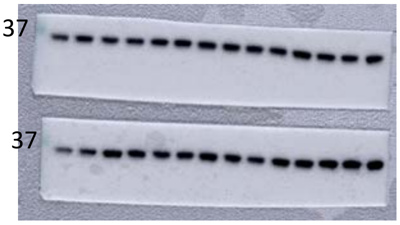

Sample order :
top : PM - N * E * N * E * N * E * N E
bottom : PM - N * E * N * E * N * E ***

Spinophilin (1 sec exposition)

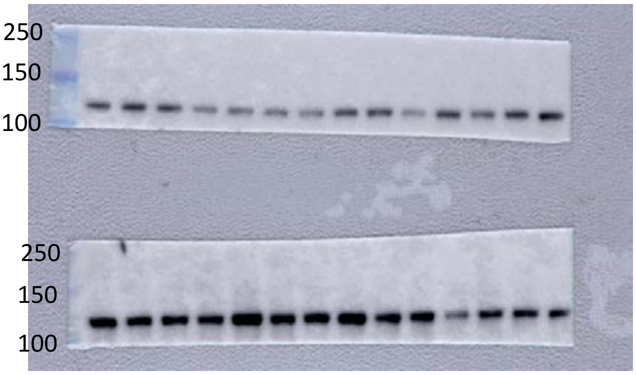

GADPH (1 sec exposition)

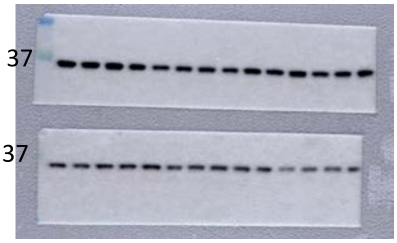

Sample order :
top : PM - N * E * N * E * N * E * N E
bottom : PM - N * E * N * E * N * E * **

nNOS (2 sec exposition)

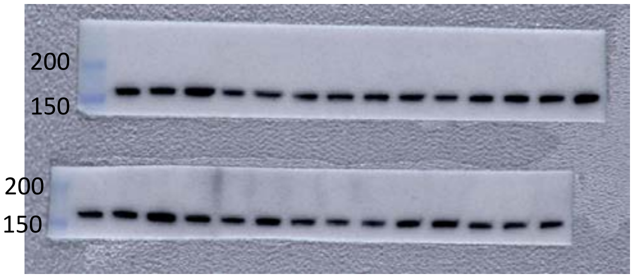

GAPDH (0.1 sec exposition)

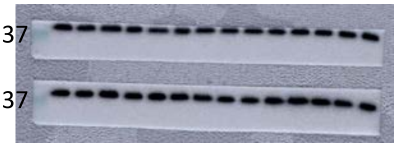

Sample order :
top : PM - N * E * N * E * N * E * N E
Bottom : PM - N * E * N * E * N * E ***

vGluT1 (1 sec exposition)

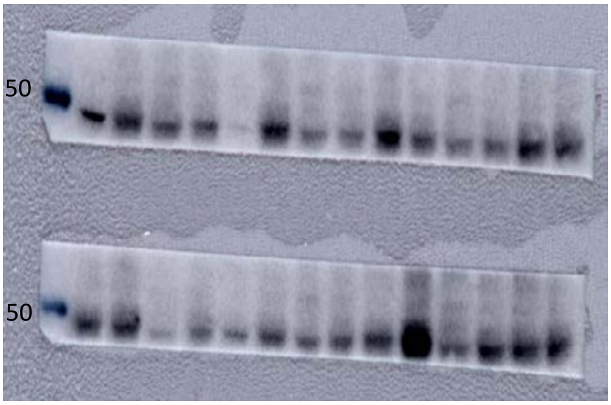

GADPH (1 sec exposition)

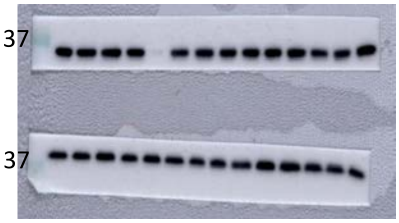

Sample order :
top : PM - N * E * N * E * N * E * N E
bottom : PM - N * E * N * E * N * E ***

vGluT2 (2 sec exposition)

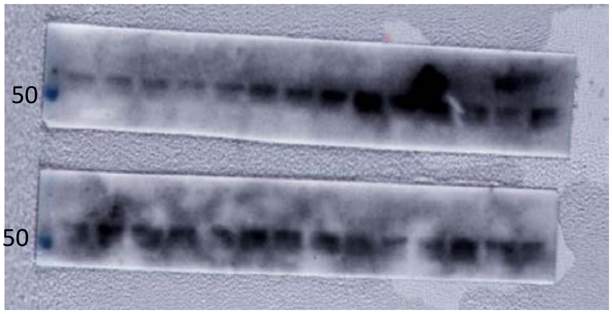

GAPDH (0.5 sec exposition)

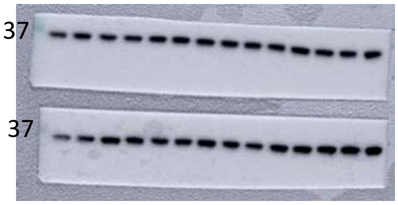

Sample order :
top : PM – N * E * N * E * N * E * N E
bottom : PM - N * E * N * E * N * E ***

GluN1 (0.8 sec exposition)

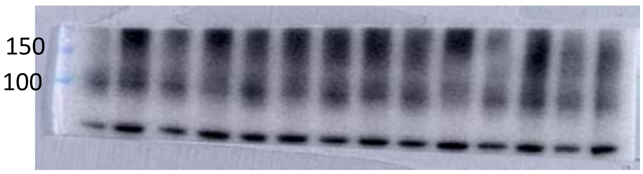

GAPDH (0.1 sec exposition)

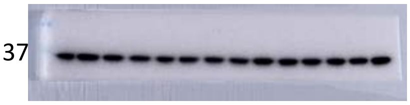

Sample order : PM - N E N E N E N E N E N E N E

GluN2A (10 sec exposition)

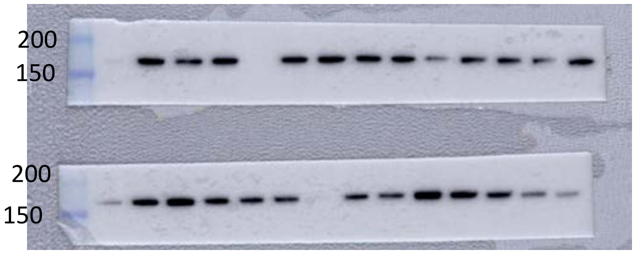

GAPDH (0.2 sec exposition)

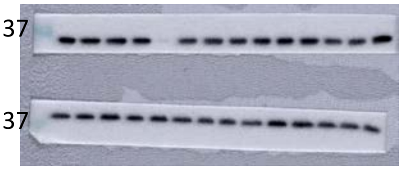

Sample order :
top : PM - N * E * N * E * N * E * N E
bottom : PM - N * E * N * E * N * E ***

GluN2B (2 sec exposition)

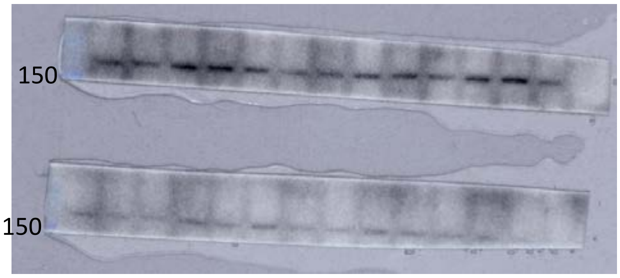

GADPH (1 sec exposition)

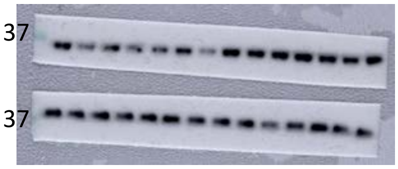

Sample order :
top : PM - N * E * N * E * N * E * N E
bottom : PM - N * E * N * E * N * E ***

GluR2 (25 sec exposition)

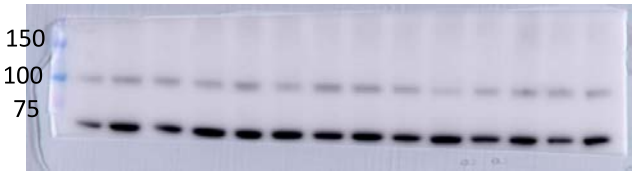

GAPDH (0.1 sec exposition)

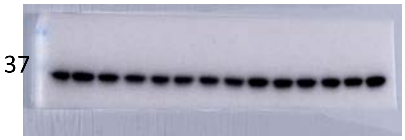

Sample order : PM - N E N E N E N E N E N E NE

Calbindin (1 sec exposition)

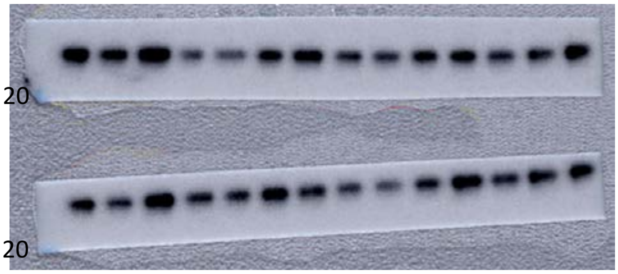

GAPDH (0.1 sec exposition)

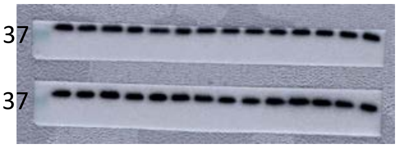

Sample order :
top : PM - N * E * N * E * N * E * N E
bottom : PM - N * E * N * E * N * E ***

GFAP (0.1 sec exposition)

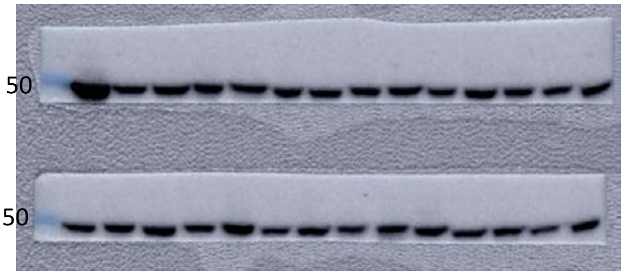

GAPDH (0.1 sec exposition)

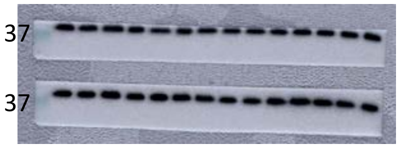

Sample order :
top : PM - N * E * N * E * N * E * N E
bottom : PM - N * E * N * E * N * E ***

GS (0.1sec exposition)

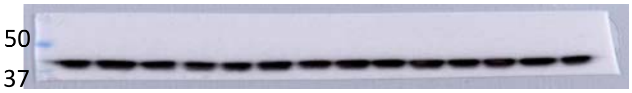

GAPDH (0.1 sec exposition)

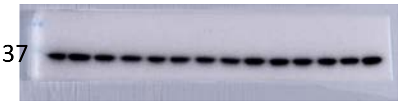

Sample order : PM - N E N E N E N E N E N E N E

GDH (4 sec exposition)

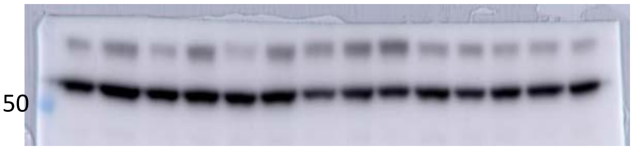

GAPDH (0.1 sec exposition)

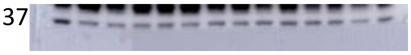

Sample order : PM - N E N E N E N E N E N E N E

Iba-1 (0.4sec exposition)

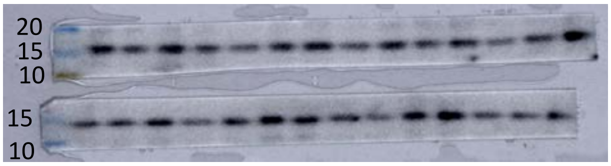

GAPDH (0.1 sec exposition)

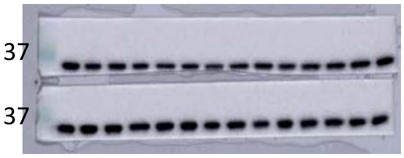

Sample order :
Top : PM - N * E * N * E * N * E * N E
Bottom : PM - N * E * N * E * N * E ***

H3ac (10 sec exposition)

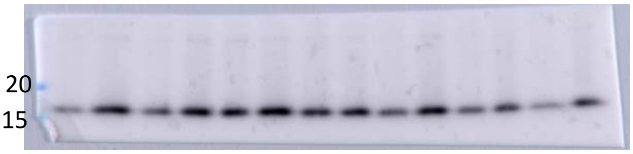

H3 (0.1 sec exposition)

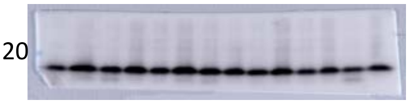

Sample order : PM - N E N E N E N E N E N E N E

H3K27me3 (5 sec exposition)

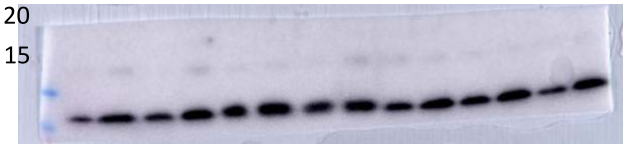

H3 (0.1 sec exposition)

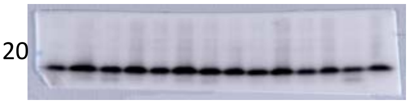

Sample order : PM - N E N E N E N E N E N E N E

H3K4me (1 sec exposition)

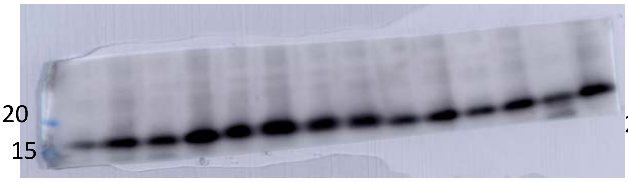

H3 (0.1 sec exposition)

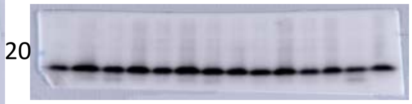

Sample order : PM - N E N E N E N E N E N E N E

H3 (0.1 sec exposition)

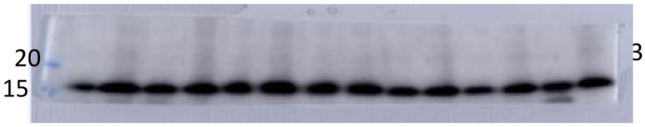

GAPDH (0.1 sec exposition)

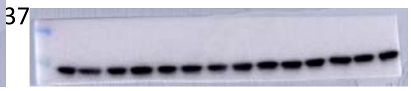

Sample order : PM - N E N E N E N E N E N E N E
